# LncRNA growth arrest specific transcript 5 inhibits the growth of pituitary neuroendocrine tumors via miR-27a-5p/cylindromatosis axis

**DOI:** 10.1080/21655979.2022.2062086

**Published:** 2022-04-17

**Authors:** Heyuan Wang, Bing Wu, Haotian Wang, Chunyan Jiang, Zhonghui Liu

**Affiliations:** a Department of Immunology, College of Basic Medical Sciences, Jilin University, Changchun, China; b Departments of Endocrinology and Metabolism, The First Hospital of Jilin University, Changchun, China; c Department of Neurosurgery, China-Japan Union Hospital of Jilin University, Changchun, China; d School of Pharmaceutical Sciences, Jilin University, Changchun, China; e NHC Key Laboratory of Hormones and Development (Tianjin Medical University), Tianjin Key Laboratory of Metabolic Diseases, Tianjin Medical University Chu Hsien-I Memorial Hospital & Tianjin Institute of Endocrinology, Tianjin, China

**Keywords:** Pituitary adenoma, lncRNA, GAS5, miR-27a-5p, CYLD

## Abstract

The long noncoding RNA growth arrest-specific transcript 5 (GAS5) has been reported to function as a suppressor in many cancers. However, the role and mechanism of lncRNA GAS5 in pituitary neuroendocrine tumors (PitNETs) remain unclear. Here, we found that lncRNA GAS5 and cylindromatosis (CYLD) expression was downregulated in invasive PitNET tissues and was negatively correlated with miR-27a-5p expression. LncRNA GAS5 overexpression inhibited proliferation of PitNETs cell line MMQ and GH3 cells and induced cell apoptosis, simultaneously, inhibited miR-27a-5p expression and increased CYLD expression. Moreover, miR-27a-5p mimic significantly decreased the luciferase activities of lncRNA GAS5 and CYLD luciferase reporter vector and downregulated CYLD expression, while miR-27a-5p inhibitor increased the expression of CYLD in MMQ and GH3 cells. Furthermore, RNA-immunoprecipitation assay revealed the direct binding between lncRNA GAS5 and miR-27a-5p. Additionally, miR-27a-5p mimic or silenced CYLD attenuated the effect of lncRNA GAS5 on MMQ and GH3 cell proliferation. In vivo lncRNA GAS5 overexpression inhibited GH3 cell tumor growth, while miR-27a-5p mimic or silenced CYLD attenuated the effect of lncRNA GAS5 on GH3 cell tumor growth. These results suggest that lncRNA GAS5 acts as an endogenous sponge by binding miR-27a-5p to increase the expression of its target gene CYLD, thereby inhibits PitNETs cell proliferation and tumor growth.

## Graphical abstract

**Figure:**
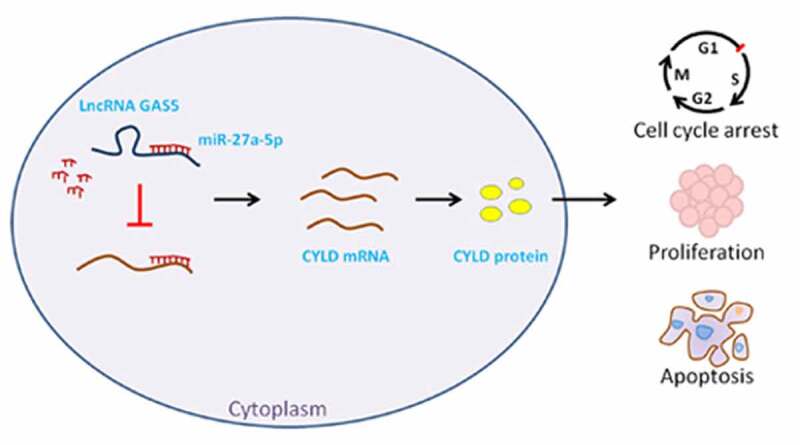

## Introduction

PitNETs, originating from cells of the pituitary gland, is a common tumor of the nervous and endocrine systems, accounting for about 8–15% of intracranial tumors [1]. The vast majority of PitNETs are benign and can be cured with surgery, medication and radiation therapy [2]. About 10% of PitNET are aggressive pituitary adenoma, which refers to a tumor exhibiting invasiveness and proliferative or clinically relevant tumor growth/recurrence despite optimal standard treatments [3]. At present, surgical resection is still the main clinical treatment for PitNETs. However, many patients with PitNETs are at risk of tumor recurrence [4]. Therefore, studying the molecular mechanism involved in unusual rapid growth of PitNETs is of great significance for discovering novel therapeutic targets and improving patient survival.

Recently, lncRNAs have been identified to participate in various physiological and pathological processes [5,6], and play crucial regulatory roles in PitNETs by exerting tumor-suppressive or oncogenic activities [7,8]. LncRNA GAS5, which was originally found to accumulate in growth-arrested cells, has been reported to serve as a tumor suppressor gene in many cancers, and its expression was often downregulated in various cancers, and its overexpression inhibited tumorigenesis, tumor progression and drug resistance [9–11]. Several studies have indicated that lncRNA GAS5 sponged miR-34a to activate the mTOR/SIRT1 pathway, thereby restraining the progression of colorectal cancer [12], and upregulated lncRNA GAS5 reduced cisplatin resistance in non-small cell lung cancer [13]. However, the possible role and mechanism of lncRNA GAS5 in PitNETs remain uncharted.

In this study, we investigated the role and mechanism of lncRNA GAS5 in PitNET tissues and cells. We found that the expression of lncRNA GAS5 was downregulated in invasive PitNET tissues, and overexpression of lncRNA GAS5 inhibited the proliferation, induced cell cycle arrest, and apoptosis of PitNET cells. Mechanistic studies confirmed that lncRNA GAS5 increased CYLD expression through competitive binding of miR-27a-5p, thereby promoting PitNETs progression. These findings indicate that lncRNA GAS5 may be a potential therapeutic target for PitNETs.

## Materials and methods

### Patients and tissues

Human PitNETs tissue specimens, including 18 cases of invasive and 20 cases of noninvasive PitNETs, were obtained from patients admitted to the China–Japan Union Hospital of Jilin University. The characteristics of the participants are shown in Table 1. The studies involving human participants were reviewed and approved by the Research Ethics Committee of the First Hospital of Jilin University (No. 2018–474). The patients provided their written informed consent to participate in this study.

**Table 1.** The clinical characteristics of the patients with pituitary neuroendocrine tumors

Variable	LncRNA GAS5 levels		*P* value
	Low (*n* = 19)	High (*n* = 19)	
Average age (Years)	43.5	46.8	
Gender Female Male	9 (47.4%) 10 (52.6%)	10 (52.6%) 9 (47.4%)	1.000
Types PRL-PA GH-PA	6 (42.9%) 13 (54.2%)	8 (57.1%) 11 (45.8%)	0.737
Knosp I II III IV	0 (0.0%) 2 (15.4%) 7 (100%) 10 (90.9%)	7 (100.0%) 11 (84.6%) 0 (0.0%) 1 (9.1%)	0.000
Ki-67 <3% 1–3% 3–5% >5%	1 (9.1%) 0 (0.0%) 9 (100.0%) 9 (100.0%)	10 (90.9%) 9 (100.0%) 0 (0.0%) 0 (0.0%)	0.000

GH-PA, growth hormone pituitary adenoma; PRL-PA, prolactin pituitary adenoma.

### Cell culture

Rat pituitary adenoma MMQ and GH3 cell lines were obtained from the American Type Cell Collection (Manassas, VA, USA). Cells were routinely cultured in the complete F-12 K medium (Invitrogen, Carlsbad, CA, USA) supplemented with 2.5% fetal bovine serum (Gibco, USA) and 15% horse serum and maintained at 37 C in a humidified atmosphere of 5% CO2.

### Quantitative real-time PCR (qRT-PCR)

Total RNA of tissues or cells was isolated with Trizol (Invitrogen, MA, USA). The qRT-PCR was conducted through PrimeScriptTM RT Master Mix and SYBR® Premix Ex TaqTM II (Takara, Shiga prefecture, Japan) using the Bio-Rad CFX96 PCR System (Bio-Rad, CA, USA). The primers were synthesized by Invitrogen, the primer sequences are listed in Table 2. The relative expression level was calculated with the 2^−ΔΔCt^ method with GAPDH or U6 as a normalization control.

**Table 2.** The sequences used in this study

The primer sequences of RT-qPCR
LncRNA GAS5 F: 5’ TTTCCCTGGGACAGATTGCC 3’ R: 5’ GTTTCATAGGCCCCTGTGCT 3’
CYLD F: 5’ AGCTAAACACTGCACCCG 3’ R: 5’ CCAGCGAGCACTTCATTC 3’
GAPDH F: 5’ ATGACATCAAGAAGGTGGTGAAGCAGG 3’ R: 5’ GCGTCAAAGGTGGAGGAGTGGGT 3’
miR-27a-5p F: 5’ TGCGG AGGGCTTAGCTGCTTGT 3’ R: 5’ CCAGTGCAGGGTCCGAGGT 3’
U6 F: 5’ GCTCGCTTCGGCAGCACA 3’ R: 5’ AACGCTTCACGAATTTGCGTG 3’
**Primer sequences for lncRNA GAS5 cloning** F: 5’-GAGGATCCCCGGGTACCGGTCGCCACCTTTCGAGGTAGGAGTCGACTCCTG-3’ R: 5’-CACACATTCCACAGCTAGTGGATTGCAAAAATTTATTAAAATTG-3’

### Cell transfection

The pcDNA3.1 vectors subcloned with the whole sequence of lncRNA GAS5 were constructed, and the primer sequences of cloning are listed in Table 2. The pcDNA3.1 vectors containing CYLD full sequence were obtained from Genechem (Shanghai, China). For overexpression of lncRNA GAS5 and CYLD, 20 nmol/L empty vectors or 100 nmol/L subclonal pcDNA3.1 vector containing the whole sequence of lncRNA GAS5 or CYLD were transfected into cells using Lipofectamine 3000 with a working concentration of 24 µL/mL (Invitrogen, Carlsbad, CA, USA). The cells with stable and high expression of lncRNA GAS5 were selected by G418 resistance screening and subcloning. For knockdown of lncRNA GAS5 and CYLD, shRNAs specifically targeting lncRNA GAS5 (sh-GAS5) or CYLD (sh-CYLD) and their negative control shRNAs (sh-NC) were transfected into MMQ and GH3 cells. miR-27a-5p mimic, inhibitor, and negative controls were synthesized and purchased from Thermo Fisher Scientific (USA). The MMQ and GH3 cells were transfected with 25 pmol/L aliquots of either the miR-27a-5p mimic or miR-27a-5p inhibitor.

### CCK-8 assay

Transfected cells were seeded into 96-well plates at the density of 1 × 104 cells/well, and incubated for 24, 48, and 72 h. Ten microlitres of CCK-8 solution (Yeasen, China) was added to each well and then incubated for 2 h. The medium was then removed and the plates were washed twice. The optical density was measured at 450 nm using a microplate analyzer (Thermo Fisher, CA, USA).

### Colony formation assay

Colony formation assay was performed according to the previous study [14]. Transfected cells were counted and seeded into 24-well plates at 500 cells per well. After incubation for 2 weeks, cells were washed, fixed in 4% paraformaldehyde for 15 min, and stained with 0.1% crystal violet solution for 30 min. Cell colonies were photographed. Colonies with more than 50 cells were counted by ImageJ software (version 1.8.0; National Institutes of Health).

### Cell cycle and apoptosis analyses

The procedure of cell cycle and apoptosis analyses were conducted following to the previous studies [15]. At 48 h after transfection, cells were harvested and resuspended in cold PBS for analysis. For cell cycle analysis, cells were stained with propidium iodide (KeyGEN Biotech, Nanjing, China) following the manufacturer’s instructions. The rate of cell apoptosis was determined using an Annexin V-FITC/PI Apoptosis Detection kit (KeyGEN Biotech, Nanjing, China) according to the manufacturer’s protocol.

### Western blot

Protein specimens were diluted in loading buffer and denatured at 95°C. The total protein (40 μg) was electrophoresed on 10% sodium dodecyl sulfate-polyacrylamide gel electrophoresis (SDS-PAGE), and transferred onto the polyvinylidene difluoride membrane. Five percent skimmed milk was used to block the membrane at room temperature for 2 h. The membrane was incubated with the primary antibodies anti-CYLD, anti-Bax, anti-Bcl-2, anti-cleaved-caspase 3, and anti-GAPDH (Abcam, USA) overnight at 4°C, followed by washing and incubation with corresponding secondary antibodies for 2  h. At length, protein samples were subjected to an enhanced chemiluminescence detection system (Bio-Rad, USA). Anti-CYLD antibody (ab137524), anti-Bax antibody (ab263897), anti-Bcl-2 (ab196495), anti-cleaved-caspase 3 antibody (ab32042) and anti-GAPDH antibody (ab8245) were purchased from Abcam (Shanghai, China).

### RNA immunoprecipitation (RIP) assay

The RIP assay was conducted as previously described [16]. The Magna RIP RNA-Binding Protein Immunoprecipitation Kit (Millipore, USA) was used following the manufacturer’s instruction. In brief, PitNET cells grown to 70–80% confluence were harvested and lysed in RIP buffer. Next, cell lysate was co-immunoprecipitated with anti-Ago2 (Sigma-Aldrich, MO, USA), or anti-IgG antibodies bound to sepharose beads. Finally, the coprecipitated RNAs were purified and used for qRT-PCR analysis.

### Dual-luciferase assay

The dual-luciferase assay was performed as previously described [17]. The wild-type (WT) or mutant lncRNA GAS5 or CYLD 3’ UTR containing the binding sites with miR-27a-5p was cloned into pmirGLO luciferase vector (Promega, Madison, WI, USA) separately. Cells on 24-well plates were co-transfected with the wild-type or mutant plasmids and miR-27a-5p mimics or miR-NC using Lipofectamine 3000. The luciferase activity was measured with a Dual-Luciferase Reporter Assay System (Promega, USA).

### Tumor xenograft model

Six-week-old male BALB/c nu/nu mice were obtained commercially from the National Laboratory Animal Center (Beijing, China). GH3 cells with stable GAS5 expression or parallel control were subcutaneously injected into the left flank of mice (five mice per group). The length (L) and width (W) of the tumors were measured every week, and tumor volume was calculated as L × W^2^ × 1/2. Mice were sacrificed on the fourth week after implant. Tumors were excised and weighed. The animal study was reviewed and approved by the Animal Research Ethics Committee of Jilin University.

### Statistical analysis

SPSS 22.0 software (SPSS Inc., Chicago, IL, USA) was used for statistical analyses, and the data from at least three independent experiments were expressed as mean ± SEM. The normal distribution of data was determined by the Shapiro–Wilk test. The difference between two groups was analyzed by a two-tailed Student’s *t*-test, while multigroup comparison was performed by one-way analysis of variance. Correlation between two variables was measured using Pearson correlation. *P* < 0.05 was considered to be statistically significant.

## Results

In this study, we investigated the role and mechanism of lncRNA GAS5 in PitNET tissues and cells. We found that the expression of lncRNA GAS5 was downregulated in invasive PitNET tissues, and overexpression of lncRNA GAS5 inhibited the proliferation, induced cell cycle arrest, and apoptosis of PitNET cells. Mechanistic studies confirmed that lncRNA GAS5 increased CYLD expression through competitive binding of miR-27a-5p, thereby promoting PitNETs progression. These findings indicate that lncRNA GAS5 may be a potential therapeutic target for PitNETs.

### LncRNA GAS5 expression was downregulated in invasive PitNETs

**Figure 1.**
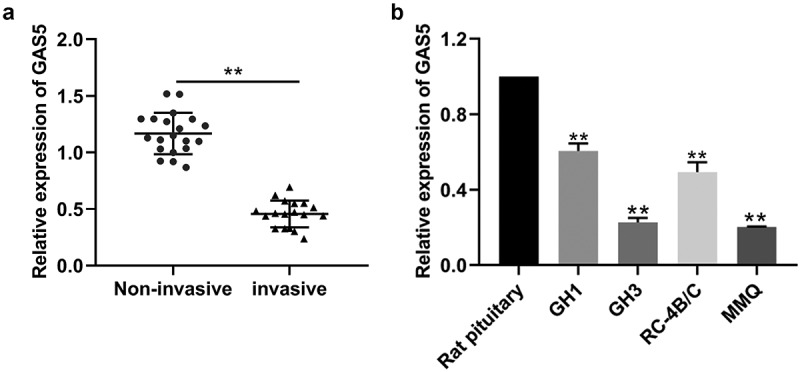
LncRNA GAS5 expression was downregulated in invasive PitNETs. (a) The levels of lncRNA GAS5 expression in tissues of invasive PitNETs (*n* = 18) and noninvasive PitNETs (*n* = 20) in humans were examined by qRT-PCR. **p < 0.01. (b) The levels of lncRNA GAS5 expression in rat PitNET cells were determined by qRT-PCR (n = 3). **p < 0.01, compared with the pituitary.

The levels of lncRNA GAS5 expression in invasive PitNET tissues and noninvasive PitNETs were examined by qRT-PCR. As shown in Figure 1a, the expression of lncRNA GAS5 was down-regulated in invasive PitNET tissues, compared with that in noninvasive PitNET tissues (*P* < 0.05). Next, we explored the relationship between lncRNA GAS5 levels and the clinical characteristics in 38 PA patients, as listed in Table 1. It was found that PA patients with GAS5low had higher Knospo invasiveness (p = 0.000) and Ki-67-positive rate (p = 0.000), and there was no significant difference between GAS5 levels and tumor types (The cutoff value is the median of the 38 PA patients, Table 1) Therefore, we investigated the prognostic implication of lncRNA GAS5 expression. Additionally, the levels of lncRNA GAS5 expression were also down-regulated in PitNETs cell lines GH1, GH3, RC-4B/C, and MMQ (Figure 1b). Of which, GH3 and MMQ cells exhibited lower levels of lncRNA GAS5 and were chosen for further experiments.

### Overexpression of lncRNA GAS5 repressed cell proliferation, induced cell cycle arrest, and apoptosis

**Figure 2.**
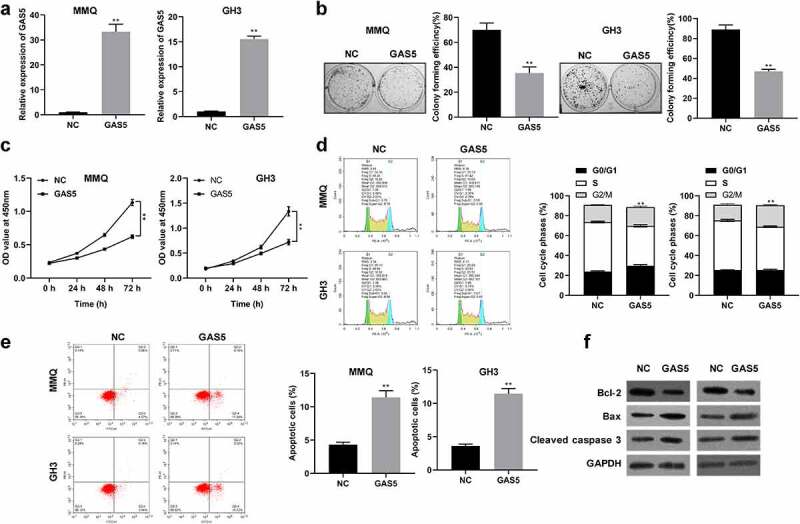
Overexpression of lncRNA GAS5 inhibited proliferation and facilitated apoptosis of PitNET cells. MMQ and GH3 cells transfected with pcDNA3.1-lncRNA GAS5 (GAS5) or empty vector (NC). (a) qRT-PCR assay was utilized to investigate the expression of lncRNA GAS5 in MMQ and GH3 cells. (b and c) The effect of lncRNA GAS5 overexpression on MMQ and GH3 cell proliferation was examined by colony formation assay and CCK8 method. (d and e) Cell cycle and cell apoptosis were determined by flow cytometry in MMQ and GH3 cells transfected with pcDNA3.1-lncRNA GAS5. (f) The protein levels of BCL-2, Bax, and cleaved caspase 3 were analyzed by Western blotting in MMQ and GH3 cells transfected with pcDNA3.1-lncRNA GAS5. **p < 0.01, compared with NC control.

To investigate the biological function of lncRNA GAS5 in PitNET cells, lncRNA GAS5-overexpressing plasmid or empty vectors were transfected into MMQ and GH3 cells. The results showed that transfection with lncRNA GAS5-overexpressing plasmid effectively increased the levels of lncRNA GAS5 expression in MMQ and GH3 cells (Figure 2a). CCK8 assay and colony formation assay showed that lncRNA GAS5 overexpression significantly inhibited cell viability and colony formation (Figure 2b, 2c). Furthermore, flow cytometry analysis implied that expression of lncRNA GAS5 caused a marked accumulation at G0/G1 phase (Figure 2d) and increased the apoptosis rate of MMQ and GH3 cells (Figure 2e). Western blotting analysis further revealed that lncRNA GAS5 overexpression increased levels of Bax and cleaved caspase 3 and decreased BCL-2 levels (figure 2f), indicating increased cell apoptosis.

In addition, we also established the lncRNA GAS5 knockdown systems by using lncRNA GAS5 siRNAs (si-GAS5) in GH1 cell lines (Figure S1A). As shown in Figure S1B, colony formation assay showed that the colony numbers were much more in both si-GAS5#1 and si-GAS5#2 groups than in the si-NC group. CCK8 assay revealed that knockdown of lncRNA GAS5 markedly increased GH1 cell viability (Figure S1C). In addition, flow cytometry analysis showed that knockdown of GAS5 reduced apoptosis rate of GH1 cells (Figure S1D). Overall, these results demonstrated that lncRNA GAS5 inhibited proliferation, but promoted apoptosis of pituitary adenoma cells.

### LncRNA GAS5 acted as a sponge for miR-27a-5p

**Figure 3.**
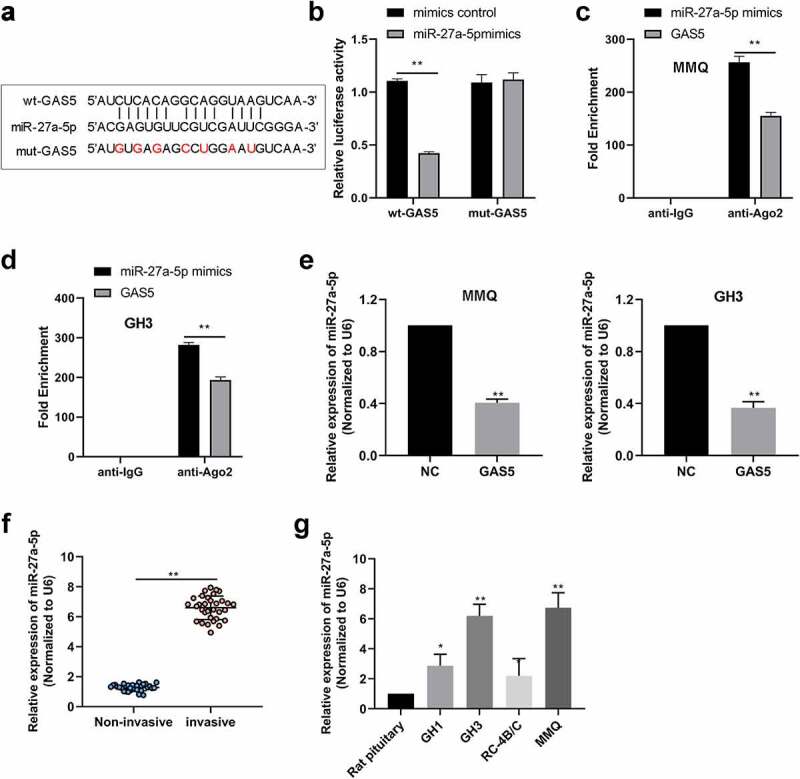
LncRNA GAS5 interacted with miR-27a-5p. (a) The interaction sites between wt-lncRNA GAS5 and miR-27a-5p were analyzed by using RegRNA website. (b) The interacting activities between lncRNA GAS5 and miR-27a-5p were examined by luciferase reporter assay. (c and d) RIP assay confirmed the interaction between lncRNA GAS5 and miR-27a-5p in MMQ and GH3 cells. (e) The levels of miR-27a-5p in MMQ and GH3 cells transfected with pcDNA3.1-lncRNA GAS5 (GAS5) were determined by qRT-PCR. (f) Relative miR-27a-5p levels in human PitNET tissues were detected by qRT-PCR. (g) Relative miR-27a-5p levels in rat PitNET cells were assayed by qRT-PCR. *p < 0.05; **p < 0.01.

Increasing evidence revealed that lncRNA GAS5 functions as competing endogenous RNAs (ceRNAs) through sponging miRNAs to prevent miRNAs from binding with their target mRNAs [11,18]. RegRNA 2.0 database (http://regrna2.mbc.nctu.edu.tw/) was used to identify that lncRNA GAS5 harbored one conjectural binding site of miR-27a-5p (Figure 3a). To determine the relationship between miR-27a-5p and lncRNA GAS5, WT lncRNA GAS5 containing the binding site of miR-27a-5p (wt-GAS5) or mutant lncRNA GAS5 (mut-GAS5) was inserted into the luciferase reporter vector (Figure 3a). The results indicated that miR-27a-5p mimic significantly decreased the luciferase activity of wt-GAS5 vector, but failed to alter that of the mut-GAS5 vector (Figure 3b). Moreover, the RIP assay showed that lncRNA GAS5 and miR-27a-5p were abounded in the Ago2 pellet (Figure 3c and 3d), further validating their binding potential. Moreover, we examined whether lncRNA GAS5 could regulate miR-27a-5p levels in PitNET cells and found that lncRNA GAS5 overexpression inhibited miR-27a-5p expression in MMQ and GH3 cells (Figure 3e). Next, we examined the expression of miR-27a-5p in PitNET tissues using qRT-PCR. We found that miR-27a-5p was distinctly up-regulated in the invasive PitNET tissues compared to noninvasive PitNET tissues (figure 3f). Furthermore, in contrast to rat pituitary, miR-27a-5p was expressed at a high level in PitNET cells (Figure 3g). These results suggested that lncRNA GAS5 could sponge miR-27a-5p.

### miR-27a-5p could directly target CYLD

**Figure 4.**
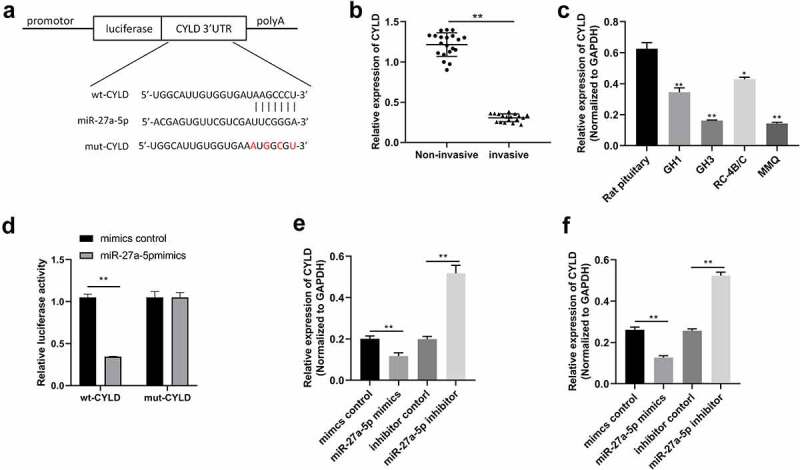
miR-27a-5p directly targeted CYLD. (a) The predicted miR-27a-5p binding sites in CYLD mRNA were revealed using Targetscan, and the mutated 3'UTRs of CYLD mRNA are shown. (b) Relative CYLD levels in human PitNET tissues were examined by qRT-PCR. (c) Relative CYLD levels in rat PitNET cells were determined by qRT-PCR. (d) The binding relation between CYLD and miR-27a-5p was assessed by luciferase reporter assay. (e) CYLD mRNA levels were detected by using qRT-PCR in MMQ and GH3 cells transfected with miR-27a-5p mimics or inhibitors. *p < 0.05; **p < 0.01.

Using the TargetScan database (http://www.targetscan.org/vert_71/), we noted that CYLD was a potential target of miR-27a-5p (Figure 4a). It was found that CYLD was decreased in invasive PitNET tissues compared to noninvasive PitNET tissues (Figure 4b). Additionally, the expression of CYLD was down-regulated in PitNETs cell lines GH1, GH3, RC-4B/C, and MMQ, compared with that of normal pituitary (Figure 4c). Further, CYLD-expressing plasmids were transfected into MMQ cells to upregulated the expression of CYLD (Figure 2SA). Then, we found that CYLD overexpression inhibited cell viability and colony formation, and promoted cell apoptosis in MMQ cells (Figure 2SB-D). Therefore, CYLD was selected as a putative target of miR-27a-5p for further analysis. To verify the putative binding site for miR-27a-5p in the 3'UTR of CYLD mRNA, we constructed vectors containing either wild-type CYLD 3'UTR (wt-CYLD) or mutant CYLD 3'UTR (mut-CYLD). As shown in Figure 4d, the relative luciferase activity of the wt-CYLD vectors was inhibited by miR-27a-5p mimics in MMQ and GH3 cells, while the relative luciferase activity of the mut-CYLD vectors was not inhibited. Furthermore, miR-27a-5p mimics decreased the expression of CYLD, whereas miR-27a-5p inhibitor increased the expression of CYLD in MMQ and GH3 cells (Figure 4e). These results indicated that CYLD may be a direct target of miR-27a-5p and can be inhibited by miR-27a-5p in PitNET cells.

### LncRNA GAS5 inhibited cell proliferation via the miR-27a-5p/CYLD axis

**Figure 5.**
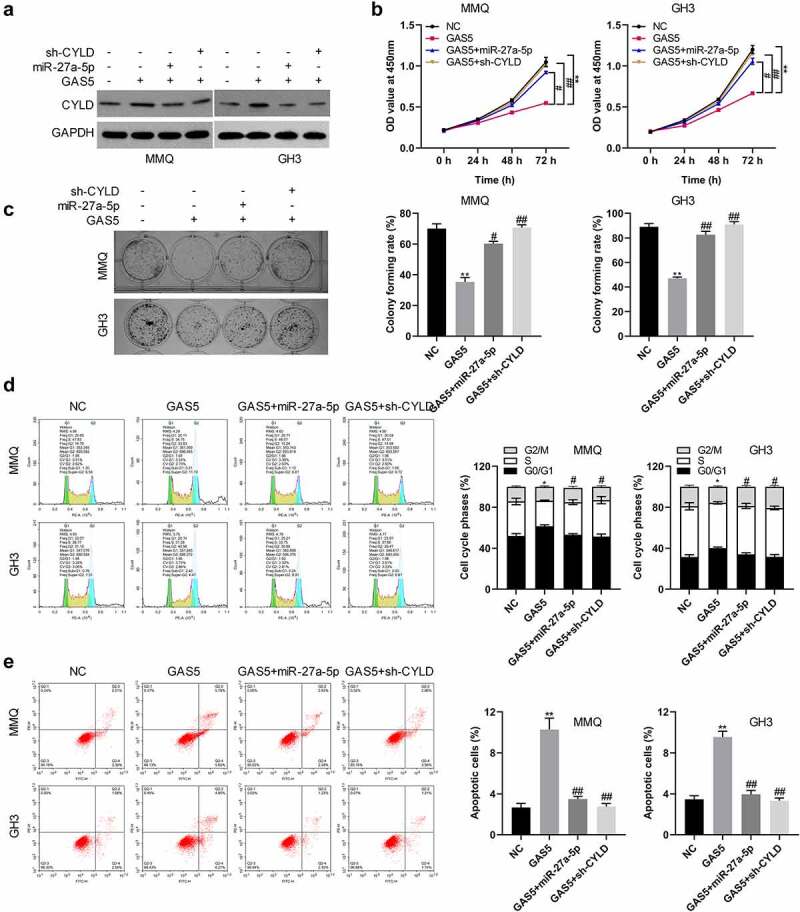
LncRNA GAS5/miR-27a-5p/CYLD axis facilitated cell proliferation in PitNET cells. (a) Western blotting was performed to examine the protein levels of CYLD in MMQ and GH3 cells after transfection of pcDNA-lncRNA GAS5 or co-transfection of miR-27a-5p mimics or sh-CYLD. (b and c) Cell proliferation was analyzed by colony formation assay and CCK8 method in lncRNA GAS5-overexpressed MMQ and GH3 cells co-transfected with miR-27a-5p mimics or sh-CYLD. (d and e) Flow cytometry assay was performed to examine cell cycle and cell apoptosis in lncRNA GAS5-overexpressed MMQ and GH3 cells co-transfected with miR-27a-5p mimics or sh-CYLD. *p < 0.05, **p < 0.01, vs. NC groups; #*p* < 0.05, ##*p* < 0.01, vs. lncRNA GAS5+ mimics-NC groups.

Based on the above results, we hypothesized that lncRNA GAS5 affects CYLD expression through inhibiting miR-27a-5p. MMQ and GH3 cells were transfected with the pcDNA3.1-lncRNA GAS5, pcDNA3.1-lncRNA GAS5 + miR-27a-5p mimic, or pcDNA3.1-lncRNA GAS5 + sh-CYLD. As expected, lncRNA GAS5 significantly increased the expression of CYLD, whereas the effect of lncRNA GAS5 on CYLD was abolished by overexpression of miR-27a-5p (Figure 5a). Moreover, CCK8 and colony formation assays showed that lncRNA GAS5 inhibited cell viability and colony formation, while miR-27a-5p mimics or silenced CYLD could attenuate the effect of lncRNA GAS5 on cell viability and colony formation (Figure 5b and 5c). Furthermore, cell cycle and cell apoptosis assays showed that lncRNA GAS5 induced G0/G1 phase cell cycle arrest and apoptosis, while miR-27a-5p mimics or silenced CYLD attenuated the effect of lncRNA GAS5 on cell cycle and cell apoptosis (Figure 5d and 5e). These results indicate that lncRNA GAS5 regulates cell activities by contradicting the inhibitory effects of miR-27a-5p on CYLD.

### LncRNA GAS5 suppressed tumor growth in vivo

**Figure 6.**
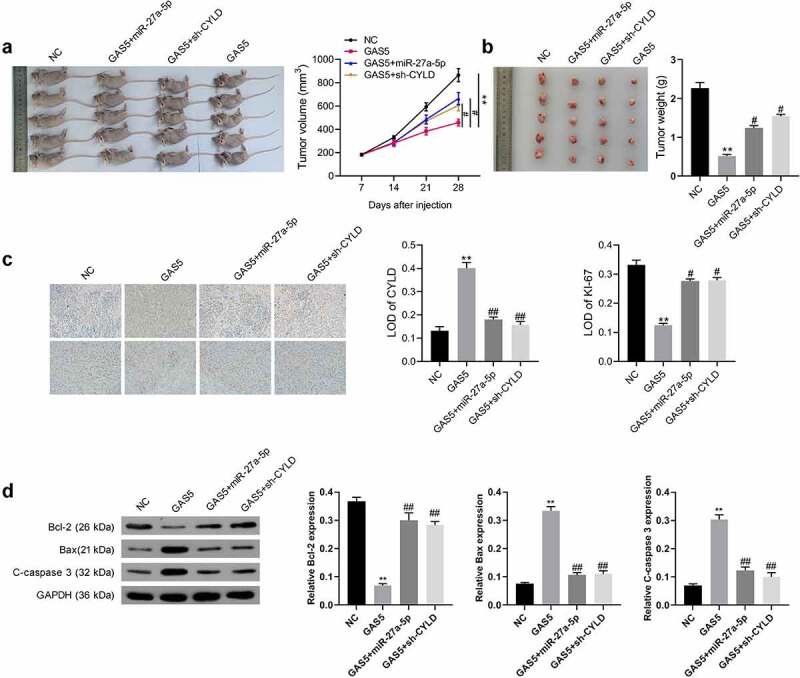
LncRNA GAS5 inhibited the tumor growth of PitNET cells via miR-27a-5p/CYLD axis in vivo. LncRNA GAS5-overexpressing GH3 cells were co-transfected with miR-27a-5p mimics or sh-CYLD. (a) Transfected cells were subcutaneously injected into nude mice (*n* = 5). (b) Tumor growth curves of each group were drawn for 28 days. (c) The tumor mass was removed and weighed 28 days after injection with indicated cells. (d) CYLD and Ki-67 expression in the indicated tissues was examined by immunohistochemical staining. **p < 0.01, vs NC groups; #*p* < 0.05, ##*p* < 0.01, vs lncRNA GAS5+ mimics-NC groups.

To further certify lncRNA GAS5 action, lncRNA GAS5-overexpressing GH3 cells were co-transfected with miR-27a-5p mimics or sh-CYLD vectors. The transfected cells were subcutaneously injected into nude mice (Figure 6a. The results indicated that tumors from the lncRNA GAS5-transfected GH3 cells grew more slowly than those from the control group during the entire tumor growth period, while miR-27a-5p mimics or silenced CYLD attenuated the effect of lncRNA GAS5 on tumor growth (Figure 6b). Accordingly, compared with the control group, tumor weight in the GAS5 overexpression group showed a significant decrease after 4 weeks, while either miR-27a-5p overexpression or sh-CYLD could reverse the inhibitory effect of GAS5 on tumor weight (Figure 6c). In addition, immunohistochemical staining showed lncRNA GAS5 overexpression caused increased CYLD and reduced Ki-67 expression, which was attenuated by either miR-27a-5p mimics or sh-CYLD (Figure 6d).

## Discussion

A large number of studies have reported that lncRNAs play extremely important roles in the occurrence and progression of tumors. LncRNAs not only act as proto-oncogenes to promote tumor formation but also as tumor suppressor genes that inhibit tumor cell proliferation and migration. LncRNA GAS5 is a member of the 5’ terminal oligo-pyrimidine class of genes and is associated with cellular growth arrest and apoptosis [19,20]. Downregulation of lncRNA GAS5 occurs in many cancers including breast cancer, non-small cell lung cancer, and colorectal cancer [9,10,21]. In this study, we found that the expression of lncRNA GAS5 in invasive PitNETs was higher than that in noninvasive PitNETs, suggesting that lncRNA GAS5 may play important roles in the tumorigenesis and progression of PitNETs. Previous studies have reported that lncRNA GAS5 was a tumor suppressor and overexpression of lncRNA GAS5 inhibited tumor progression by activating YAP phosphorylation [21–23]. LncRNA GAS5 was also recognized as the important driver of chemoresistance in many cancers, upregulated lncRNA GAS5 enhanced chemosensitivity and cell apoptotic death of triple-negative breast cancer cells, and inhibited DDP-resistance through the lncRNA GAS5-E2F4-PARP1-MAPK axis in epithelial ovarian cancer [24–26]. These findings suggest that lncRNA GAS5 inhibits tumor progression in a variety of ways. In the present study, we demonstrated lncRNA GAS5 as a tumor suppressor suppressed proliferation of PitNET cells, and overexpressed GAS5 caused cell cycle arrest and apoptosis of PitNET cells. These data indicate that lncRNA GAS5 exerts an anti-cancer role in PitNETs.

The mechanism of lncRNA action is very complex, among which ceRNA is the most common action mode of lncRNA in the cytoplasm. LncRNAs carry seed sequences of certain miRNAs that sponge miRNAs and prevent miRNAs from binding to target genes [27,28]. Our data indicated that lncRNA GAS5 was a sponge of miR-27a-5p and overexpression of lncRNA GAS5 significantly inhibited the expression of miR-27a-5p. We also verified a direct association between miR-27a-5p and CYLD. Other studies have reported that lncRNAs acted as a sponge of miRNAs during the tumorigenesis and progression of PitNETs [29,30], and the lncRNA CLRN1-AS1 sponged miR-217 to promote the dickkopf WNT signaling pathway inhibitor 1 (DKK1) and regulate cell activities in pituitary prolactinoma [31].

Many studies confirmed that miRNAs play important roles in PitNETs tumorigenesis and progression [32,33]. miR-27a-5p was shown to be significantly downregulated in prostate tissues, along with aberrant promoter methylation, while its overexpression in PC3 cells inhibited cell growth, suggesting a tumor-suppressive role for miR-27a-5p [34]. Our results provided evidence that miR-27a-5p was a direct downstream target of lncRNA GAS5 via luciferase assay. Herein, overexpression of lncRNA GAS5 in PitNET cells decreased the expression of miR-27a-5p, thereby inhibiting proliferation, promoting G0/G1 phase cell cycle arrest and apoptosis of PitNET cells.

*CYLD* gene is a tumor suppressor gene reported in 2003, which exists widely in human tissues. As a deubiquitination enzyme, CYLD plays a crucial regulatory role in the cell cycle and tumorigenesis inhibition by inhibiting JNK, NF-KB, WNT, and other signaling pathways [35]. Some studies have shown that CYLD can inhibit cancer cell proliferation, migration, invasion, and metastasis, and downregulated CYLD is associated with the development and progression of cervical cancer [36]. In this study, we found that the expression of CYLD in invasive PitNETs was significantly lower than that in noninvasive PitNETs. Further studies showed that CYLD was the regulatory target of lncRNA GAS5/miR-27a-5p. Overexpression of lncRNA GAS5 or miR-27a-5p inhibitor promoted CYLD expression. Moreover, the luciferase assay confirmed that miR-27a-5p was directly bound to CYLD 3 ‘UTR region, and the expression of CYLD negatively correlated with miR-27a-5p in PitNET tissues. In addition, this study also demonstrated that lncRNA GAS5 overexpression significantly increased CYLD expression in vivo. These findings suggest that lncRNA GAS5 protects CYLD from miR-27a-5-mediated CYLD mRNA degradation through ceRNA-mediated fashion in PitNET cells.

## Conclusion

This study revealed that lncRNA GAS5 regulated PitNET cell activities through a reciprocal interaction with miR-27a-5p, and overexpression of GAS5 inhibited cell proliferation and induce cell apoptosis by affecting the cell cycle in PitNET cells. These findings indicate that lncRNA GAS5 is a tumor suppressor and used for therapeutic intervention of PitNETs in the future.

## Supplementary Material

Supplemental Material

## Data Availability

The datasets used and/or analyzed during the current study are available from the corresponding author on reasonable request.
