# Supplementary figures and images for: LncRNA growth arrest specific transcript 5 inhibits the growth of pituitary neuroendocrine tumors via miR-27a-5p/cylindromatosis axis
Source: Bioengineered. 2022 Apr 17;13(4):10274–86. doi: 10.1080/21655979.2022.2062086 (PMC9162025; doi:10.1080/21655979.2022.2062086)

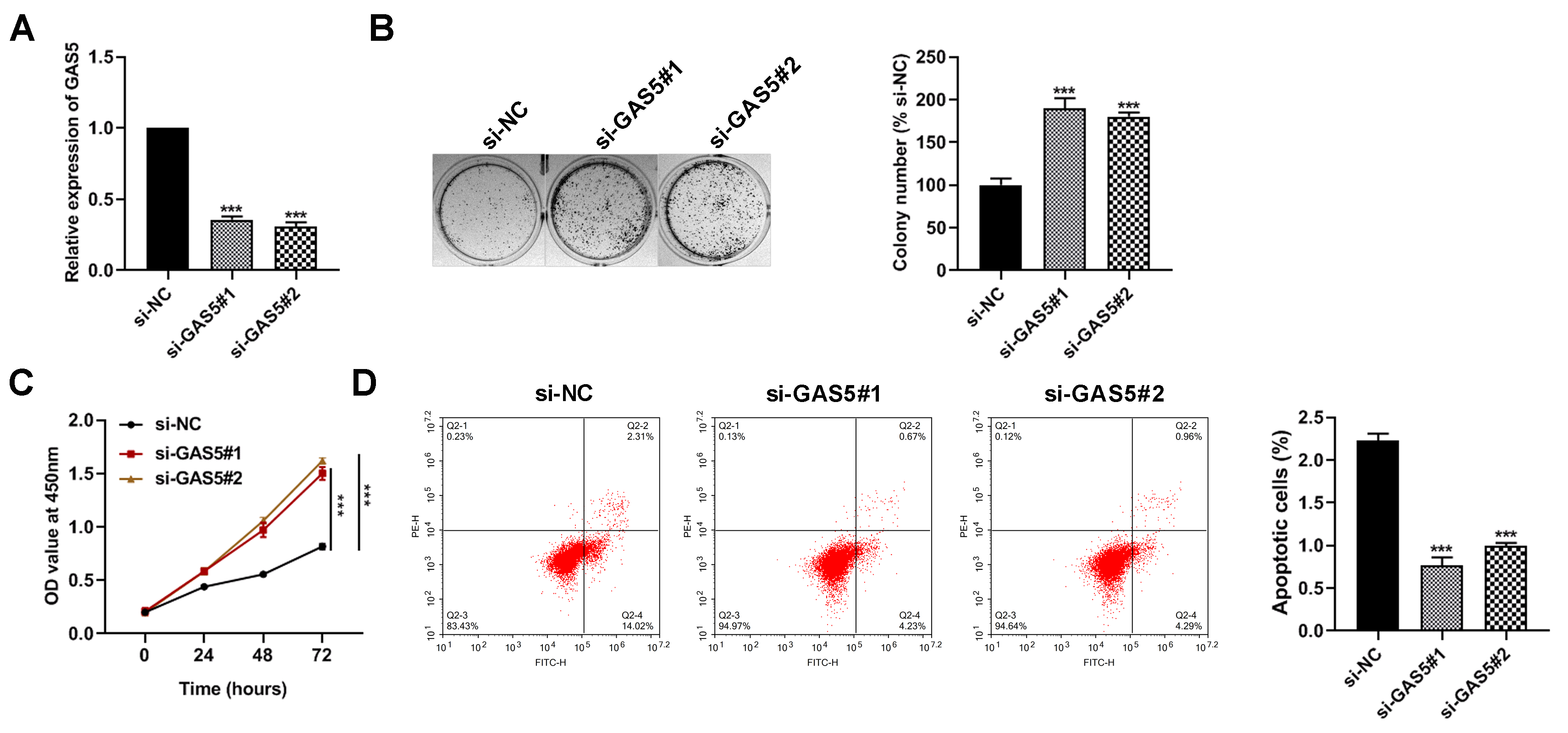

Supplement: Supplemental Material [file KBIE_A_2062086_SM3223.zip › supplementary/Figure S1.tif]

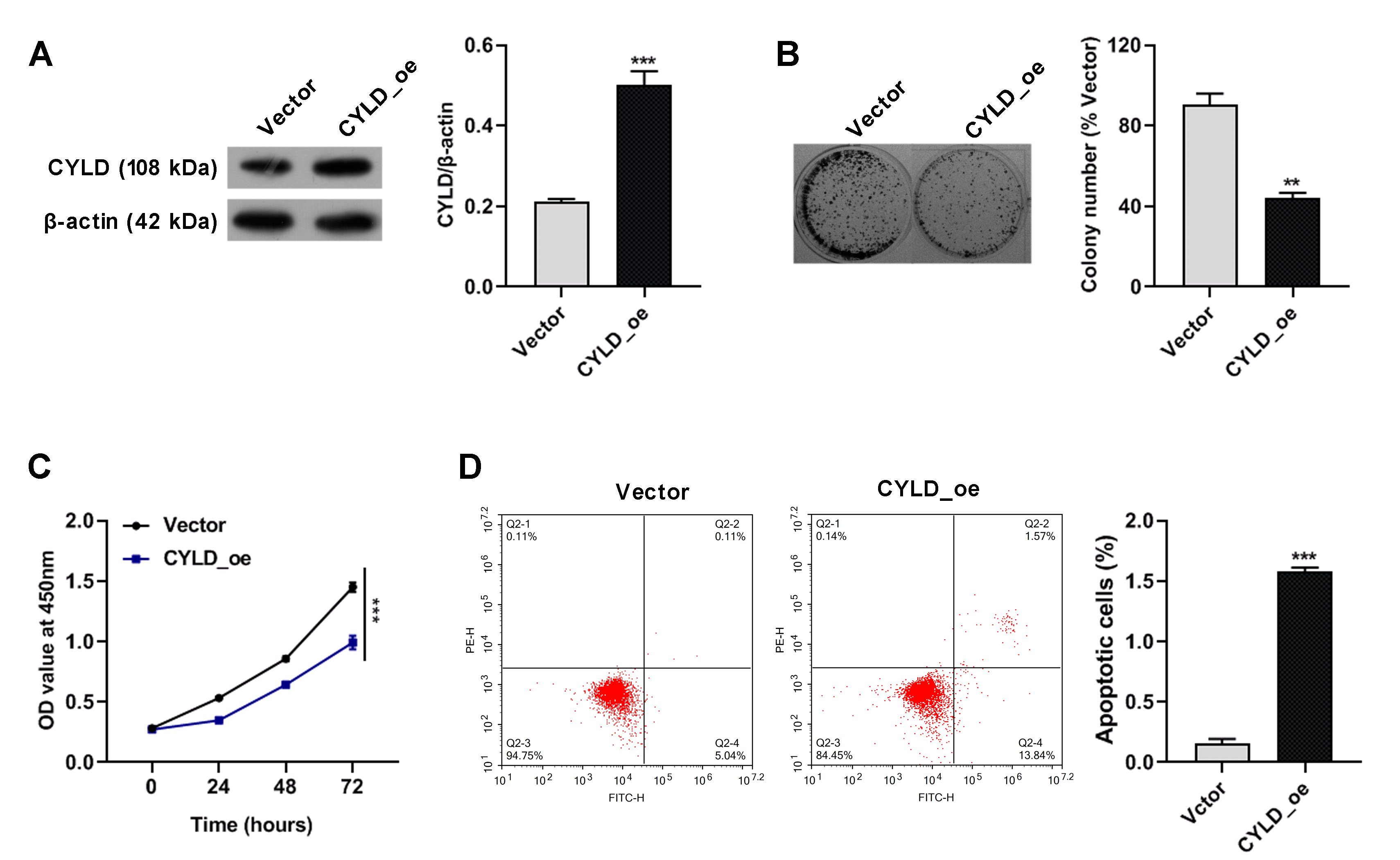

Supplement: Supplemental Material [file KBIE_A_2062086_SM3223.zip › supplementary/Figure S2.tif]
